# Matching middle and top managers: Do gender and tenure similarities between middle and top managers affect organizational performance?

**DOI:** 10.1371/journal.pone.0249246

**Published:** 2021-03-26

**Authors:** Kornelis F. van den Oever

**Affiliations:** Nijmegen School of Management, Radboud University Nijmegen, Institute for Management Research, Nijmegen, The Netherlands; Bucharest University of Economic Studies, ROMANIA

## Abstract

This paper studies whether demographic similarities between middle and top managers with different tasks (strategy formation and strategy implementation) impacts organizational performance. By drawing on relational demography theory, we investigate the effect of similarity in gender, organizational tenure, and in both these demographics on the overall costs of Dutch municipalities. The main findings of this paper show that the similarity effects are interrelated: when middle and top managers diverge on only one demographic, performance is increased. Also, when leaders are similar on both demographics, performance is impaired. We conclude by discussing the implications for the literature on middle management, relational demography, and strategy formation and implementation.

## Introduction

The notion that strategy affects firm performance lies at the heart of strategic management research [1]. For firm performance, it is paramount that strategy formation and implementation are matched [2]. Where top managers are mainly responsible for strategy formation, the main tasks of middle management is strategy implementation [3, 4], yet middle management also provides inputs in the strategy formation process [3, 5]. As such, the interaction between top managers and middle managers is crucial in effective strategy formation and implementation [6].

Research has shown that the alignment between the characteristics of top managers and middle managers is important, e.g. in the implementation of management innovation [7]. However, the literature on strategy formation and implementation has been relatively silent on the role of the match of top managers and middle managers, even though we know from the relational demography perspective that individuals’ supportive behavior towards others is affected by how similar individuals are to each other [8, 9]. As such, the match between top managers and middle managers can hinder or support the adequate implementation of formulated strategy.

In this paper, we apply the relational demography perspective by studying whether similarities on a biodemographic (gender) and a functional characteristic (organizational tenure) affect organizational performance. We also examine how the effects of these similarities may be contingent on one another, i.e. similarity on both characteristics may amplify the individual similarity effects on performance. Formally, we pose the research question: *“To what extent does similarity in gender and/or tenure between middle and top managers affect organizational performance*?*”* By focusing on the similarity of characteristics of top managers and middle managers, we attempt to shed new light on the psychological foundations of middle management behavior, as called for by Wooldridge, Schmid [10].

We study this question in the Dutch local government setting, which provides an excellent arena to study our research question. First, in these organizations, there is a specific division of strategy formation and implementation tasks, where the city manager is in charge of strategy implementation, and the aldermen team is responsible for strategy formation [11]. This allows us to specifically analyze the match between these individuals and to relate this to organizational performance. Second, a common problem for strategic leadership studies is that the composition of these teams is endogenously determined [12]. In our setting, the city’s aldermen team is predominantly formed from and by the city’s council, which are elected. The city manager is selected by the aldermen and mayor, just as middle managers are selected by top managers in firms. However, in our setting, this selection of middle managers needs to be approved by the works council. As such, the top management team does not have full control over who will be the middle manager, and as such the match between middle and top management is less susceptible to endogeneity issues.

This research intends to contribute to the literature in multiple ways. First, we intend to extend studies on relational demography by showing that gender and tenure similarities between leaders responsible for strategy implementation and formation are specifically important for organizational performance. Although similarity on these separate dimensions can be beneficial for the organization, when similarity exists across multiple dimensions, we find that the organization suffers. As such, we contribute to the relational demography literature by showing that similarity across multiple dimensions may actually be adverse.

Second, we contribute to the literature on interfaces between middle and top management [6], by specifically studying how similarities between middle and top management affect organizational performance. Although the middle management literature has drawn attention to the importance of strategy implementation by organizational leaders [10, 13], the match between strategic leaders who implement and who form strategies has been underemphasized. Our paper intends to empirically show this match’s importance to explain organizational outcomes.

## Theory and hypothesis development

Top and middle management play important roles in strategy formation and implementation. Middle managers are defined as occupants of the hierarchical layer “between the operating core and the apex” [14: 98]. Although top management is mostly formally responsible for strategy formation and middle management for its implementation [3, 4, 15, 16], we know from the middle management literature that middle management has a large influence on the formation of strategy as well [13]. Middle managers encourage their followers to engage in experimentation and generate new ideas [5, 17–19]. They may relax procedures and formal controls in order to promote such divergent activity [3, 17, 20]. Towards top management, middle managers contribute to organizations’ strategy formation by raising new strategic initiatives that would otherwise not be considered [e.g., 21]. This role has been described as their strategic championing role ─ defined as “the persistent and persuasive communication of strategic options to upper management” [3: 155].

In their role as implementers of strategy, middle managers deviate in their commitment to adequately execute their task, where some even outright resist to implement a strategy if middle and top management’s interests are not aligned [4, 22]. Although strategy implementation may seem straightforward, the details of the strategy need to be operationalized, which occurs through interpretation and a sensemaking process of middle managers [23–25]. Increased involvement by middle managers in the strategy formation can enhance implementation as it increases understanding of and commitment to the strategy [13]. For adequate strategy implementation, it is thus important that middle managers have adequate understanding of the strategy as it is intended by top management, which is gained through interaction between middle and top management [6]. The relational demography perspective can help elucidate when such understanding and commitment is more pervasive.

### Relational demography

In theorizing how similarity in characteristics between individuals affects their supportive or non-supportive behavior, relational demography has been specifically influential. Relational demography is defined as “the comparative demographic characteristics of members of dyads or groups who are in a position to engage in regular interactions” [9: 403]. Relational demography proposes that an individual’s difference or similarity in their demographics to others affects the attitudes and behavior of the individuals toward each other, which affects employment outcomes [26]. The main tenets of this perspective are based on the similarity-attraction paradigm, which states that individuals who are similar in attitudes or personal characteristics will be attracted to each other [27–29].

Using this theoretical lens, evidence has been built that shows that demographically similar employees like and prefer to work with one another [30], demographical similarity increases higher levels of social integration [31], increased communication [32], a greater desire to remain [8], more positive affect and commitment [33], enhanced group functioning [34], and lower turnover [35]. Employees view demographically similar others as more reliable, trustworthy, and competent [36], and dissimilar others as more dishonest, untrustworthy, and uncooperative [37]. Such similarity in demographics is also shown to drive selection of board members in entrepreneurial firms [38]. Also, in employee-customer interactions, being similar leads to fewer customer complaints [39].

As such, the relational demography literature points towards a prediction in which similarity between middle management and top management is beneficial. before we discuss how similarity in gender and organizational tenure is beneficial for the performance of the organization, we briefly take stock of the fundamental assumptions we make in this paper. First, a core assumption in relational demography is that individuals prefer to be with similar others [8]. Second, we assume that top and middle management frequently interact [13], as otherwise their relational demographic characteristics would have limited effect. Third, we assume that the individuals studies in relational demography (e.g., on supervisor-subordinate relationships) can be applied to top manager-middle manager relations as well, where we note that relational demography has already been applied to within top management relationships [40].

### Gender similarity

Gender stereotypes are widely shared and reinforced via a range of social interactions—more so because gender is a highly visible, stable, and ascriptive characteristic [41, 42]. Individuals use these attributions to make up for incomplete information about others, and rely on such stereotypes in understanding others’ viewpoints [43]. Because of these mechanisms, individuals are evaluated by their peers on whether the content of their contributions is in line with prevalent gender stereotypes. When individuals do not behave in accordance to these stereotypes, they are negatively evaluated [42]. As individuals will seek to prevent getting a negative evaluation, this may lead to female and male executes and directors acting in accordance to their stereotypes.

Indeed, individuals that are different in gender also have different ideas and perspectives and give priority to different issues [44–46]. Although this may be helpful in joint decision-making [47], if strategic leaders do not necessarily jointly make decisions, these differences in ideas and perspectives and issues attended to can actually materialize in their actions. For example, women have been found to focus more on philanthropy and community service than men [48]. Thus, if the individual that is formulating the strategy is a woman, she may believe that philanthropy and the community are important to include in the organization’s strategy. If the individual that is executing the strategy is a man, he may believe that these issues are not as important, thus executing a strategy that is incongruent with the one formulated.

In addition, same-sex superiors and subordinates experience more positive working relationships [49]. Likewise, women in upper echelon positions create a culture that is friendly to women and committed to the advancement of women at all levels [50, 51], stimulating interaction between top and middle management levels that are similar in gender. Tsui and O’Reilly [9] find empirical evidence of these mechanisms and showed that differences in gender between individuals correlated with more negative performance appraisals and affect between the individuals.

These mechanisms stand to be particularly problematic for middle and top managers that differ between one another, especially when they have different fundamental but interrelated goals, such as strategy formation and implementation. When there is a decreased frequency of communication and less affect between such individuals, it becomes less likely that the execution of strategy will follow the formulation, as the person responsible for execution will have lesser opportunities to understand the strategy as it is formulated. Likewise, the leaders that formulate the strategy will have fewer conversations with the leaders involved with execution, such that the strategy formulated may become disjointed from the day-to-day business of the organization. In such case, since a match between strategy formation and implementation is beneficial for organizational performance, gender dissimilar strategic leaders will be less able to ensure appropriate organizational performance. We thus hypothesize:

*H1*: *Greater gender similarity between middle management and top management leads to increased organizational performance*.

### Organizational tenure similarity

Alike to gender, organizational tenure similarity stimulates interaction between individuals. For instance, Zenger and Lawrence [32] found that the more engineers’ tenure differed from others in their organization, the less the engineers communicated with these colleagues. Indeed, interpersonal workplace relationships stemming from tenure similarity have been argued to be stronger than those deriving from any other characteristic [52]. Over and above the general relational demography mechanisms, differences in organizational tenure also have their own unique processes.

Individuals that have similar organizational tenure have both recently started at the organization, have been working for a long time at the organization, or something in-between. Strategic leaders that have just started have led to strategic changes of the organization as it allows leaders to overcome organizational inertia [53–55]. Leaders that are longer in their position are more likely to resist change as they have more power to resist such change [56, 57]. Such power arises from hiring other individuals in the organization that have similar ideas as the longer tenured leader. Leaders with shorter tenures have not been able to put in place individuals in the organization with similar ideas, so for these leaders it is more difficult to resist change.

As such, strategic leaders with similar organizational tenure, are likely to be like-minded. Either they are receptive to change the strategy, in case when they have low tenure, or may be hostile to strategy change, when they have high tenure. This implies that leaders with similar tenure will also have more similar ideas and perspectives on strategy, making it more likely that strategy formulation and execution are matched. Thus, when there is an increasing organizational tenure similarity between leaders that are responsible for strategy execution and formulation, the organization is likely to have higher organizational performance. We thus, based on this and earlier mechanisms, hypothesize:

*H2*: *Greater organizational tenure similarity between middle management and top management leads to increased organizational performance*.

### Combined similarity in gender and organizational tenure

Although relational demography proposes that similarity on single characteristics increases attraction between two individuals, we theorize further how similarity on multiple characteristics could influence attraction. As gender and organizational tenure are reflective of a person’s attitudes or values [58], similarity on each characteristic can increase attractiveness. The values and attitudes of an individual are not simply an additive of the characteristics of a person, a combination of characteristics can uniquely identify a person’s values and attitudes. For instance, men care more about power, security, conformity, and tradition [59]. Their focus on tradition may make them less likely to vouch for changes when their organizational tenure is high, compared to women who emphasize inclusion [60] and may break with tradition. Hence, in the combination of characteristics, the values and attitudes of a person manifest.

As individuals become more alike in their values and attitudes, they become more attracted to one another [27–29], further increasing interaction [34, 61, 62]. Given the increased interaction because of the combined similarity in gender and organizational tenure, strategy implementation is more likely to be aligned with the formed strategy by top management [6]. Hence, we hypothesize:

*H3*: *Greater similarity on gender and organizational tenure between middle management and top management leads to increased organizational performance*.

## Methodology

We study our research question in the context of Dutch municipalities. Dutch municipalities are, after the national government and the provinces, the third layer of government in the Netherlands. Its organization and governance is vested in the Municipality Law of 1994. The municipality is tasked with the execution of national policy, but they also have the authority to form their own policy regarding urban development, traffic and transportation, education, welfare and social affairs, and taxation.

The city’s council is the highest hierarchical level in the municipality. Its members are elected every 4 years by public elections. The board size of the council, which ranges from 9 to 45, is dependent on the number of inhabitants. Their tasks are primarily to formulate the frameworks in which the strategy should be formulated, engaging in decision control regarding the council of mayor and aldermen, and formally deciding on the budget.

The council of mayor and aldermen carry out the day-to-day management of the municipality. They are primarily responsible for the finance and budget of the municipality. Given the frameworks stipulated by the city’s council, they form the strategy of the municipality. The aldermen are determined by the city’s council. This is done after public elections for the board, where political parties engage in a coalition formation process. Those parties that are in the coalition typically select one or two of their leaders to become aldermen, although aldermen do not necessarily need to be affiliated with a party. These aldermen remain in office until the new elections, which are held every four years. In our sample, elections occurred three times (in 2006, 2010, and 2014). Each alderman has his or her own specialty, e.g. education, finance, sport and culture. Every municipality needs at least two aldermen, but cannot have more than 20% of the number of council members. In our sample, the average number of aldermen was 3 per municipality. The mayor is elected by the Crown and is primarily responsible for public order and safety and is otherwise engaged in ceremonial activities. Given this, in our study we primarily look at the demographics of the aldermen and how they relate with the city manager.

Next to the city’s council and the council of mayor and aldermen is the formal organization with civil servants. Civil servants prepare decisions to be made by the council of mayor and aldermen and execute the decisions made. The city manager heads the civil servants and is the connection between the formal organization and the council of mayor and aldermen. The city manager is primarily responsible for the adequate implementation of the strategy formed by the council of mayor and aldermen [11]. As such, a focus on the aldermen and the city manager provide an excellent research setting to study our research question.

### Data collection

We collected our data from multiple public sources. For the data on organizational performance and the various control variables we used data from the Dutch Central Bureau of Statistics. For the data on the tenure and gender of aldermen and city managers, we collected the data from www.overheidinnederland.nl, which contains information on the composition of the various strategic leadership teams, including the gender and organizational tenure of the individuals involved. In total, the number of organization-year observations is 720, with 198 unique organizations.

### Dependent variable

*Organizational performance*_*i,t*+1_ We measure performance by considering the total costs per inhabitant in euros (from Central Bureau of Statistics), or simply put, the budget deviation. Although municipalities’ performance hinges on more dimensions of performance than cost efficiency, the broad concepts of efficiency and effectiveness are generally held to be applicable to them [63, 64]. Furthermore, a growing number of countries face a declining trend in economic growth that creates fiscal pressure or “austerity” conditions [65]. As a result, cost efficiency becomes a critical goal for public organizations [65, 66]. Hence, we deem total costs per inhabitant to be an adequate performance variable for this organization. We lead this variable by one year to make better causal inferences.

### Independent variables

*Gender similarity*_i,t_. We measured gender similarity by constructing a dummy variable. First, we considered the number of male aldermen and number of female aldermen in organization *i* at time *t*. When there were more female aldermen than male aldermen, and the city manager was female, the dummy variable would be 1. If there were more male aldermen than female aldermen and the city manager was male, the dummy variable would also be 1. In all other cases, the dummy variable would be 0.

*Organizational tenure similarity*_i,t_. To measure organizational tenure similarity, we used a similar approach as Heyden, Sidhu [7]. First, we calculated the organizational tenure of the city manager, which was the number of years since the city manager was active as city manager in organization *i* at year *t*. Then, we calculated the average aldermen organizational tenure by calculating the average number of years since all aldermen were active as aldermen in organization *i* at year *t*. We then applied the following formula:

$$\begin{array}{l} {Organizational\;tenure\;similarity}_{i,t}\\ \;=|{Organizational\;tenure\;city\;manager}_{i,t}\\ \;-{Average\;aldermen\;organizational\;tenure}_{i,t}|*-1 \end{array}$$

We multiplied by -1 so that an increase in this variable can be interpreted as an increase in organizational tenure similarity.

### Control variables

We controlled for several factors. First, we controlled for the budgeted total costs per inhabitant in euros. Including the budgeted total costs allows us to control for factors that affect budgeted total costs (e.g. the tendency of certain political parties to cut costs). We lead this variable since the budget is made one year prior to the budgeted year. Second, we controlled for gender of the city manager with a dummy variable that equals one if the city manager was a woman, as this may affect the city manager’s aptitude and leeway given by others in the organization to implement strategies. Third, we controlled for the organizational tenure of the city manager by calculating the number of years since the city manager was active as city manager in organization *i* at year *t*. Longer tenured city managers may be more able to better implement strategies as they have better knowledge about the role and of the organization. Fourth, since the mayor is another important leader in our setting who may influence strategy formation and execution, the gender of the mayor was taken as a control variable, which equals one if the mayor was a woman. Fifth, and for similar reasons, we controlled for the organizational tenure of the mayor by calculating the number of years since the mayor was active as mayor in organization *i* at year *t*. Sixth, we used the aldermen gender diversity measure through calculating Blau’s heterogeneity index as a control, as more gender diverse top management teams can foster greater municipality performance [67]. Seventh, average aldermen organizational tenure, as specified above, was used as a control variable. This was control was added for similar reasons as we control for aldermen gender diversity. Eight, we used the total number of individuals living in municipality *i* at year *t* as a control, given that the scale of the municipality can impact its efficiency. Ninth, to account for economic differences, we used the average value of property in euro’s as a control. Tenth, to further account for economic differences, we measured the number of jobs per 1,000 inhabitants. Twelfth, we included year dummies and *municipality fixed effects*.

### Analysis

We employed the following fixed effects OLS (ordinary least squares) model:

$$\begin{array}{l} {Organizational\;performance}_{i,t+1}\\ \;=\beta_{0}+\beta_{1}{Gender\;similarity}_{i,t}+\beta_{2}{Organizational\;tenure\;similarity}_{i,t}\\ \;+\beta_{3}{Gender\;similarity}_{i,t}*{Organizational\;tenure\;similarity}_{i,t}+\beta_{4}{CV}_{i,t}\\ \;+{FE}_{i}+{TE}_{t}+\varepsilon_{i,t} \end{array}$$

where *CV_i,t_* is a vector of control variables for organization *i* at time *t*, *FE_i_* denote fixed effects, *TE_t_* denote time effects, and *ε_i,t_* are idiosyncratic errors.

## Results

We checked multicollinearity and the distribution of the independent variables. Multicollinearity did arise from including the interaction term between gender similarity and tenure similarity (its VIF scores are higher than 10). Given that this is due to the interaction term, we decided to proceed to include all variables. Table 1 shows the mean, standard deviation, minimum and maximum of each variable. Table 2 provides the matrix of correlations among all variables. From these descriptives, we take note that there is sufficient variation in our dependent variable, where the average costs per inhabitant is 3,061 euro, and the standard deviation is 1,084 euro. We also note that there is limited variation in gender of the leaders (for the city manager and the aldermen team), as is generally the case when considering leadership positions. Nevertheless, the independent variables (i.e. gender and tenure similarity of leaders) show ample variation. Tenure of the different leaders is approximately 5 years, and has a considerable range as well. Furthermore, from the correlation table, we notice that there is little pairwise correlation between the independent variables and the dependent variable (ranging from -0.06 to 0.10).

**Table 1:** Descriptive statistics (n = 766).

Variable	Mean	S.D.	Min	Max
Organizational performance	-3,060.70	1,083.89	-10,131	-1,281
Gender similarity	0.69	0.34	0	1
Tenure similarity	-3.49	3.94	-24.67	0
Gender similarity * Tenure similarity	-2.82	3.96	-24.67	0
Budgeted costs	2,505.98	879.63	1,15	7,700
City Manager female	0.20	0.40	0	1
City Manager tenure	5.15	4.76	0	29
Mayor female	0.15	0.36	0	1
Mayor tenure	5.41	4.04	0	24
Aldermen gender diversity	0.17	0.21	0	0.5
Aldermen average tenure	3.77	2.25	0	19
Total population	55,098.27	77,123.19	1,103	789,270
Average value of property	243.78	62.86	116	672
Number of jobs	667.98	183.05	252.5	1,295.6
ydm2007	0.12	0.32	0	1
ydm2008	0.12	0.33	0	1
ydm2009	0.14	0.35	0	1
ydm2010	0.10	0.30	0	1
ydm2011	0.14	0.35	0	1
ydm2012	0.13	0.34	0	1
ydm2013	0.12	0.32	0	1

**Table 2:** Correlation matrix (n = 766).

	Variable	1	2	3	4	5	6	7	8	9	10	11	12	13	14	15	16	17	18	19	20	21
1	Organizational performance	1																				
2	Gender similarity	0.10	1																			
3	Tenure similarity	-0.04	-0.17	1																		
4	Gender similarity * Tenure similarity	-0.06	-0.41	0.92	1																	
5	Budgeted costs	-0.81	-0.11	0.04	0.06	1																
6	City Manager female	-0.06	-0.79	0.17	0.32	0.06	1															
7	City Manager tenure	0.06	0.21	-0.78	-0.77	-0.07	-0.21	1														
8	Mayor female	0.05	0.03	-0.03	-0.03	-0.05	-0.06	-0.02	1													
9	Mayor tenure	-0.01	0.04	-0.06	-0.08	0.02	-0.03	0.15	-0.07	1												
10	Aldermen gender diversity	-0.13	-0.43	0.05	0.20	0.16	0.02	-0.07	0.01	-0.05	1											
11	Aldermen average tenure	0.03	0.03	-0.17	-0.14	-0.02	-0.00	0.14	0.00	0.04	-0.06	1										
12	Total population	-0.57	-0.08	0.08	0.10	0.60	0.07	-0.07	-0.05	-0.05	0.13	-0.04	1									
13	Average value of property	0.22	-0.06	-0.09	-0.06	-0.21	0.00	0.07	0.05	-0.01	-0.01	-0.11	-0.14	1								
14	Number of jobs	-0.34	-0.07	-0.08	-0.06	0.37	0.06	0.09	0.00	0.00	0.10	-0.00	0.28	0.01	1							
15	ydm2007	-0.04	0.03	0.00	-0.01	-0.03	-0.05	-0.02	-0.01	-0.02	0.01	-0.12	-0.00	-0.02	-0.02	1						
16	ydm2008	-0.14	0.01	-0.00	-0.01	0.05	-0.05	-0.03	-0.00	-0.01	0.02	-0.03	-0.00	0.06	0.01	-0.10	1					
17	ydm2009	-0.04	0.02	0.01	0.00	0.08	-0.03	-0.03	-0.01	0.02	0.02	0.04	-0.00	0.10	0.02	-0.10	-0.10	1				
18	ydm2010	-0.04	0.01	0.01	-0.00	0.06	0.00	-0.01	0.01	-0.00	-0.04	0.08	-0.00	0.11	0.01	-0.10	-0.10	-0.10	1			
19	ydm2011	0.00	-0.04	-0.02	-0.01	0.04	0.03	-0.02	0.02	-0.02	0.06	-0.11	0.00	0.08	0.02	-0.10	-0.10	-0.10	-0.10	1		
20	ydm2012	0.05	-0.06	-0.01	0.01	0.02	0.05	-0.00	0.02	-0.01	0.05	-0.03	0.00	0.05	0.02	-0.10	-0.10	-0.10	-0.10	-0.10	1	
21	ydm2013	0.05	-0.06	0.00	0.02	0.01	0.04	0.01	-0.00	0.00	0.07	0.05	0.00	-0.00	0.01	-0.10	-0.10	-0.10	-0.10	-0.10	-0.10	1

Table 3 shows the results of the fixed effects OLS regression. Model 1 includes the control variables only. Model 2 estimates the effect of gender similarity on performance, including the control variables. In model 3, we estimate the effect of organizational tenure similarity on performance, including the control variables. In models 4 we include both the direct effects of gender similarity and organizational tenure similarity and the control variables. Finally, in model 5 we include the interaction term between gender and organizational tenure similarity. The improved R^2^ for the full model (model 5) shows that it has a superior fit.

**Table 3:** Fixed-effects OLS results.

	DV = Organizational performance									
Model	1		2		3		4		5	
	Coef.	p-value	Coef.	p-value	Coef.	p-value	Coef.	p-value	Coef.	p-value
Gender similarity (H1 +)			354.590	0.104			352.203	0.107	132.575	0.568
			(217.669)				(217.936)		(231.857)	
Tenure similarity (H2 +)					-5.440	0.675	-4.825	0.710	45.632	0.047
					(12.968)		(12.955)		(22.899)	
Gender similarity * Tenure similarity (H3 +)									-67.265	0.008
									(25.238)	
Budgeted costs	-0.376	0.000	-0.386	0.000	-0.377	0.000	-0.387	0.000	-0.371	0.000
	(0.071)		(0.072)		(0.072)		(0.072)		(0.071)	
City Manager female	-175.899	0.210	90.895	0.673	-175.379	0.212	89.561	0.678	16.790	0.938
	(140.294)		(215.511)		(140.405)		(215.712)		(216.238)	
City Manager tenure	-4.238	0.691	-2.147	0.842	-6.159	0.596	-3.865	0.741	-6.863	0.557
	(10.673)		(10.734)		(11.621)		(11.691)		(11.680)	
Mayor female	36.585	0.812	21.842	0.887	39.064	0.799	24.140	0.875	45.696	0.765
	(153.348)		(153.383)		(153.578)		(153.629)		(152.985)	
Mayor tenure	-0.069	0.994	-0.518	0.953	0.619	0.945	0.095	0.992	4.357	0.633
	(8.884)		(8.875)		(9.041)		(9.033)		(9.124)	
Aldermen gender diversity	-176.308	0.267	106.930	0.649	-170.558	0.285	110.123	0.640	204.334	0.389
	(158.550)		(235.143)		(159.261)		(235.486)		(236.824)	
Aldermen average tenure	16.023	0.210	15.732	0.218	14.879	0.255	14.720	0.260	14.347	0.269
	(12.765)		(12.747)		(13.063)		(13.044)		(12.972)	
Total population	-0.005	0.525	-0.008	0.315	-0.005	0.527	-0.008	0.317	-0.008	0.318
	(0.008)		(0.008)		(0.008)		(0.008)		(0.008)	
Average value of property	3.296	0.364	3.721	0.306	3.390	0.352	3.801	0.297	3.389	0.350
	(3.629)		(3.632)		(3.638)		(3.642)		(3.625)	
Number of jobs	-0.682	0.569	-0.786	0.512	-0.705	0.557	-0.805	0.502	-1.207	0.316
	(1.197)		(1.197)		(1.199)		(1.199)		(1.202)	
Constant	-1,962.210	0.090	-2,131.910	0.066	-1,977.610	0.088	-2,144.428	0.065	-1,693.050	0.147
	(1,154.282)		(1,157.23)		(1,155.740)		(1,158.636)		(1,164.547)	
Observations	766		766		766		766		766	
R^2^	0.211		0.215		0.211		0.215		0.225	

Municipality-fixed effects and time-fixed effects are included in all models.

To interpret the direct effects of gender similarity and organizational tenure similarity separately on organizational performance, we turn to model 4. The positive coefficient of gender similarity is consistent with hypothesis 1 (*β* = 352.203, *p* = 0.107). Yet, given the relative high p-value, we cannot claim that there is a direct effect of gender similarity on performance. Hence, hypothesis 1 is not supported.

Tenure similarity has a negative but small effect on performance (*β* = -4.825, *p* = 0.710), contrary to our hypothesis 2, which stated that tenure similarity would have a positive effect. The magnitude is small, such that one year difference in tenure between the aldermen and city manager would increase costs by 4.83 euro per inhabitant. Thus, we do not find support for hypothesis 2.

Turning to model 5, the main effects of gender similarity and tenure similarity become significant when the interaction term between them is included. The effect of gender similarity becomes more less strong (*β* = 132.575, *p* = 0.568), when organizational tenure similarity is 0 (which implies very high tenure similarity since tenure similarity has no positive values by construction). A one-unit increase in gender similarity would thus decrease the costs per inhabitant by 132.58 euro when the aldermen and city manager have equal organizational tenure. As for organizational tenure similarity, the effect becomes positive, consistent with hypothesis 2 (*β* = 45.632, *p* = 0.047) when gender similarity is 0 (which implies no similarity in gender). A one unit increase in tenure similarity thus reduces the costs per inhabitant by 45.63 euro when the aldermen and city manager have a different gender. This implies that the direct effects of gender similarity and tenure similarity are contingent upon one another, which we hypothesized in H3, although in a different manner.

**Fig 1:**
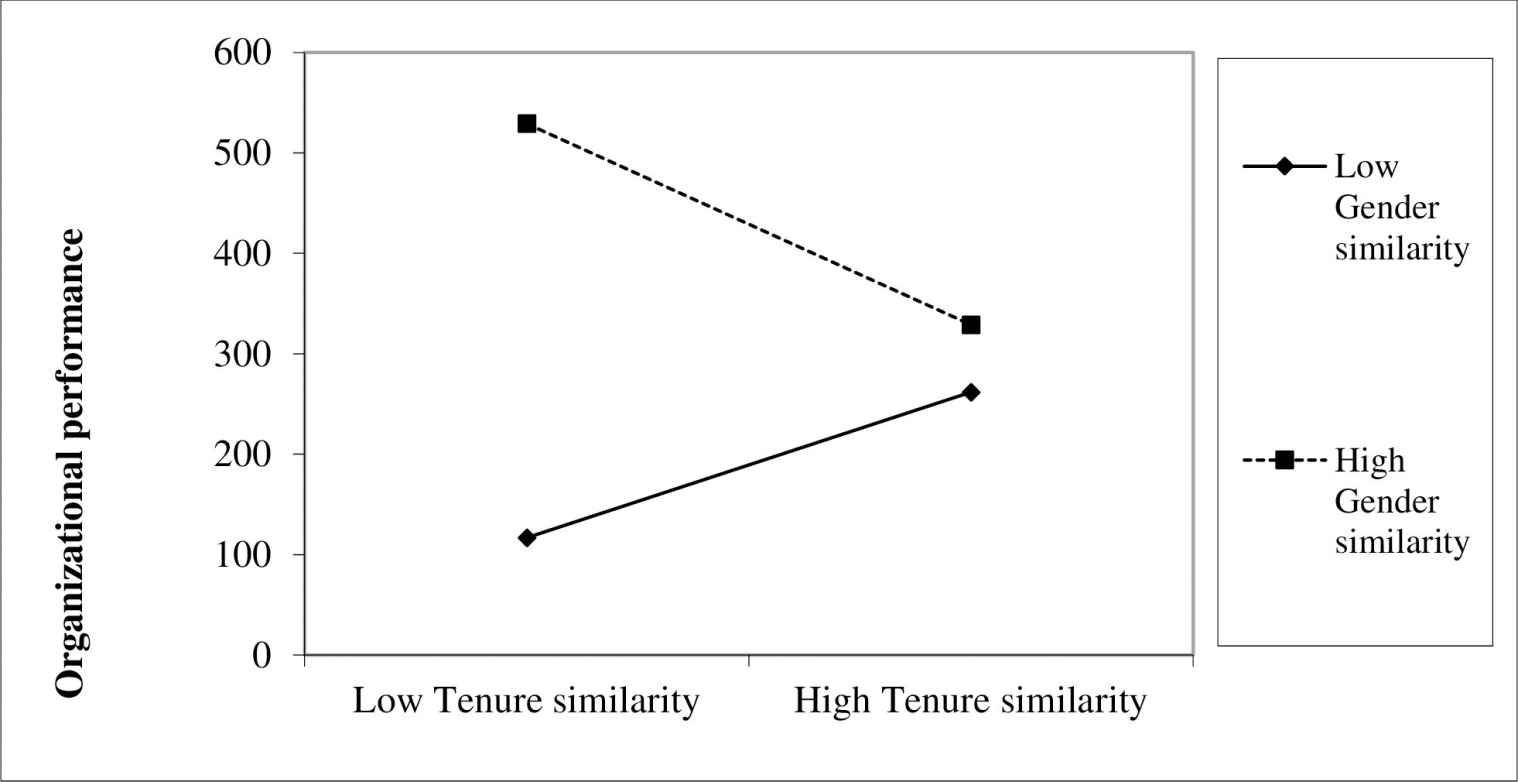
Interaction effect of tenure similarity and gender similarity.

In model 5, the interaction term between gender similarity and tenure similarity is negative and significant with a magnitude of -67.65 (p = 0.008). This is inconsistent with H3, which predicted a positive interaction effect. Fig 1 elucidates how gender similarity and organizational tenure similarity jointly affect performance. The figure shows that increasing tenure similarity is beneficial for organizational performance when gender similarity is low, but decreases performance when gender similarity is high (based on one-standard-deviation departures from the mean). Likewise, gender similarity increases organizational performance when tenure similarity is low, but has relative low impact on organizational performance when tenure similarity is high. Therefore, we also fail to find support for hypothesis 3.

### Supplemental quantitative analyses

In Table 4, we further use the D-score as an alternative measurement approach for gender similarity, by calculating the Euclidean distance (D-score) [68]:

$$D=1-\sqrt{(\frac{1}{n})-\sum {\left(S_{i}-S_{j}\right)}^{2}}$$

where *D* is the D-score, *n* is the number of city manager-alderman pairs, *S*_*i*_ the gender/tenure of the city managers, and *S*_*j*_ is the gender/tenure of alderman *j*.

**Table 4:** Fixed-effects OLS results–using D-scores for gender and tenure similarity.

	DV = Organizational performance							
Model	6		7		8		9	
	Coef.	p-value	Coef.	p-value	Coef.	p-value	Coef.	p-value
Gender similarity (H1 +)	104.219	0.432			104.616	0.431	-57.732	0.704
	(132.581)				(132.720)		(151.971)	
Tenure similarity (H2 +)			0.051	0.876	0.056	0.865	39.171	0.030
			(0.327)		(0.328)		(18.033)	
Gender similarity * Tenure similarity (H3 +)							-39.115	0.030
							(18.030)	
City Manager female	-0.378	0.000	-0.377	0.000	-0.379	0.000	-0.371	0.000
	(0.071)		(0.072)		(0.072)		(0.072)	
City Manager tenure	-103.604	0.537	-175.782	0.211	-103.202	0.539	-156.362	0.356
	(167.793)		(140.422)		(167.960)		(169.172)	
Mayor female	-3.421	0.750	-4.169	0.697	-3.343	0.756	-0.125	0.991
	(10.727)		(10.691)		(10.746)		(10.812)	
Mayor tenure	32.242	0.834	36.498	0.812	32.131	0.834	49.940	0.745
	(153.502)		(153.487)		(153.640)		(153.338)	
Aldermen gender diversity	0.250	0.978	-0.037	0.997	0.286	0.974	2.684	0.764
	(8.897)		(8.894)		(8.907)		(8.945)	
Aldermen average tenure	-83.034	0.675	-176.418	0.267	-82.799	0.676	-12.197	0.951
	(198.079)		(158.694)		(198.261)		(200.249)	
Total population	15.022	0.242	15.983	0.212	14.975	0.244	18.215	0.158
	(12.833)		(12.779)		(12.848)		(12.891)	
Average value of property	-0.006	0.481	-0.005	0.523	-0.006	0.479	-0.006	0.430
	(0.008)		(0.008)		(0.008)		(0.008)	
Number of felonies	3.444	0.344	3.308	0.363	3.458	0.342	2.948	0.418
	(3.635)		(3.633)		(3.639)		(3.634)	
Number of jobs	-0.713	0.552	-0.682	0.570	-0.713	0.552	-0.961	0.424
	(1.198)		(1.198)		(1.199)		(1.201)	
Constant	-2,031.02	0.080	-1,961.690	0.090	-2,030.710	0.080	-1,637.430	0.162
	(1,158.001)		(1,155.323)		(1,159.039)		(1,169.235)	
Observations	766		766		766		766	
R^2^	0.212		0.211		0.212		0.219	

Municipality-fixed effects and time-fixed effects are included in all models.

The results are broadly in line with those found earlier. Nevertheless, gender similarity has a different signs when using the D-score as measurement in the final model. Gender similarity is also insignificant in the final model (model 9) (*β* = -57.732, *p* = 0.704). Also, the finding that gender similarity and tenure similarity jointly affect performance is consistent with the original measurement of the two variables.

Although we noted in the introduction that the similarity between middle and top management can be treated exogenously, the council of aldermen does have the responsibility to select the city managers. As a robustness check, we ran additional models on a subsample of the dataset in which observations where the city manager was selected by the council of aldermen were excluded.

Table 5 shows that the results are consistent with those of the main results. As such, our results are robust to exclusion of the observations where the city manager started. Hence, we can rule out self-selection concerns.

**Table 5:** Fixed-effects OLS results–excluding observations where the city manager started.

	DV = Organizational performance							
Model	10		11		12		13	
	Coef.	p-value	Coef.	p-value	Coef.	p-value	Coef.	p-value
Gender similarity (H1 +)	332.393	0.164			330.077	0.167	95.528	0.709
	(238.322)				(238.215)		(256.102)	
Tenure similarity (H2 +)			18.328	0.228	18.157	0.232	67.465	0.008
			(15.194)		(15.179)		(25.407)	
Gender similarity * Tenure similarity (H3 +)							-66.566	0.016
							(27.587)	
City Manager female	-0.296	0.000	-0.279	0.000	-0.290	0.000	-0.274	0.000
	(0.078)		(0.078)		(0.078)		(0.078)	
City Manager tenure	-109.598	0.635	-309.126	0.093	-115.457	0.617	-169.338	0.463
	(230.797)		(183.776)		(230.738)		(230.584)	
Mayor female	-18.181	0.183	-13.396	0.350	-12.984	0.364	-14.529	0.308
	(13.623)		(14.304)		(14.293)		(14.230)	
Mayor tenure	124.911	0.479	141.409	0.421	121.572	0.490	127.651	0.467
	(176.213)		(175.748)		(176.150)		(175.224)	
Aldermen gender diversity	-2.562	0.783	-4.046	0.668	-4.496	0.634	-0.358	0.970
	(9.295)		(9.434)		(9.430)		(9.535)	
Aldermen average tenure	-62.018	0.803	-333.793	0.055	-84.712	0.735	-1.717	0.995
	(249.04)		(173.404)		(249.642)		(250.674)	
Total population	9.609	0.475	13.652	0.319	12.694	0.354	11.653	0.392
	(13.436)		(13.672)		(13.675)		(13.609)	
Average value of property	-0.005	0.658	0.000	0.969	-0.005	0.631	-0.005	0.622
	(0.010)		(0.010)		(0.010)		(0.010)	
Number of felonies	0.895	0.827	0.477	0.907	0.724	0.860	0.226	0.956
	(4.093)		(4.094)		(4.094)		(4.077)	
Number of jobs	-1.524	0.235	-1.353	0.292	-1.465	0.254	-1.868	0.147
	(1.283)		(1.282)		(1.283)		(1.287)	
Constant	-1,310.590	0.312	-1,258.930	0.332	-1,268.820	0.328	-780.197	0.550
	(1,295.036)		(1,296.195)		(1,294.883)		(1,303.762)	
Observations	650		650		650		650	
R^2^	0.223		0.222		0.225		0.235	

Municipality-fixed effects and time-fixed effects are included in all models.

## Discussion

We started this paper by arguing that the literature on strategy formation and implementation has been relatively silent on the match between top and middle managers. Assuming that this match would improve the strategy formed and the way they were implemented would affect organizational performance [2, 6], our findings showed that this match is indeed related to organizational performance, albeit in unexpected ways. Although our findings of direct–although not always significant–effects of similarity on gender and organizational tenure were generally aligned with work in relational demography [8, 9], our results showed an important boundary condition for these effects. That is, the effect of the degree to which individuals are similar on one dimension, is affected by the degree to which individuals are similar on other dimensions. In short, the main message is that similarity on one of two characteristics is beneficial, similarity on none and *both* characteristics is suboptimal.

One reason why too much similarity can impair organizational performance is in the nature of the individuals involved and their multiplex roles. Middle managers engage in both integrative and divergent roles [3]. The interaction resulting from similarity is expected to be especially helpful for middle managers’ integrative role, as a greater understanding of the strategy will help in implementing the strategy accordingly [2]. Yet, when individuals become too similar and engage in more and more interaction [34, 62], a limited range of divergent ideas will be explored, leading to suboptimal strategies. Characteristics, such as gender, education, and functional experience, serve as proxies for members’ information, points of view, and mind-sets [69]. When there is similarity on too many characteristics between middle and top management, middle management will be more limited in the points of view and information they can bring to top management. Indeed, this may also be the reason why we observe that the tenure of the city manager negatively impacts the performance of the organization.

In particular high gender match and low organizational tenure match proved to be most beneficial for organizational performance. The nature of the characteristics may play a fundamental role here, where similarity on a biodemographical characteristic (gender) can help in fostering a positive relationship between two individuals, dissimilarity on a functional characteristic may help individuals to engage in task-related conflict which is beneficial for the organization [69, 70]. The study of interactions of these types of characteristics may thus be helpful in understanding broader interpersonal, but also organizational outcomes. Although there is some work in relational demography that stipulates that dissimilarities can be beneficial (when considering relational norms) [71], to our knowledge, no study has studied the interaction effects of multiple characteristics. As such, this paper brings novel theoretical contributions to the relational demography literature.

This paper extends the growing literature that focuses on the interfaces between middle and top management [6]. Where Heyden, Sidhu [7] show that the alignment of characteristics of top and middle managers is important in the implementation of management innovation, we show that such alignment has even larger repercussions–affecting the overall performance of the organization. The focus on interfaces between middle and top managers, particular in how similar these managers are, also builds on and extends work on the psychological foundations of middle management behavior [as called for by 10]. Although we do not measure interaction between middle and top managers, using and building on relational demography does provide more insight in why some organizations are more efficient than others.

In general this paper also contributes to our understanding of organizational performance more generally. For organizational performance, it is paramount that strategy formation and implementation are matched [2]. Conceptually, Raes, Heijltjes [6] have advocated that the interaction between top managers and middle managers is crucial in effective strategy formation and implementation. Our paper sheds light on in which way interaction can be fostered so that top and middle managers communicate effectively to form and implement strategy. Although interaction stimulated by similarity is important, similarity on too many characteristics (especially functional characteristics) can hinder organizational performance.

### Managerial implications

The managerial implications of this study are threefold. First, as this study shows, it is important to take in consideration the demographic characteristics when selecting top and middle managers. Strategic leaders should be selected that do not differ on multiple dimensions with other important leaders whose tasks and responsibilities are interrelated. Specifically, in our setting, when selecting a city manager, top management should select an individual that is similar, but not on multiple demographic characteristics. Likewise, when the city council selects their aldermen team, they should take into account how they match with the city manager, to properly ensure that strategy execution and formulation are synchronized. Ensuring there is an appropriate match will increase organizational performance. Therefore, the seemingly political acts of leaders to promoted like-minded individuals in other powerful positions [56, 57], can in fact be beneficial for organizational performance.

Second, if middle and top managers that are not similar on any demographic dimension, they and others in the organization should be aware of the potential drawbacks they may face because of the mismatch. Although such leaders may unconsciously escape conversations, it is important to ensure that middle and top managers do meet and discuss their ideas and perspectives, especially when their tasks and responsibilities interrelate. In our setting, city managers and the aldermen team need to work together to execute and formulate strategy, in which cooperation and interaction between them is paramount. Overcoming this demographic dissimilarity bias starts with being aware of it and taking appropriate actions to ensure the bias is mitigated.

Third, specific recommendations for this setting revolve around our results that show when the city manager and aldermen team deviate on their tenure, but are similar in their gender, higher potential organizational performance can be reached. Specifically, this suggests that if a new aldermen team comes into place with no tenure, it is paramount that a longer tenured city manager is in place (and they are similar on gender). Likewise, to ensure proper organizational performance, it is important to refrain from changing the city manager when the aldermen team has low organizational tenure, as their tenure similarity will be low.

### Limitations and suggestions for future research

Notwithstanding its contributions, this study has some limitations that suggest areas for future research. One possible limitation of this study is the external validity. Municipalities are public organizations, where the tasks and responsibilities of strategy execution and formulation are clearly disentangled between individuals. Although this allowed us to better infer the mechanisms by which demography similarities impact performance, more work in other research settings is warranted. If strategy implementation and formation are not as clearly disentangled between individuals, this could force individuals to cooperate and overcome demography mismatches, however it could also exacerbate the adverse effects.

Second, not only is the external validity limited in the industry setting, but also the institutional context. For instance, the Netherlands is a country that scores particularly low on power distance, which may have implications for the results of the study. That is, as power distance decreases, top and middle managers more cooperatively work together to execute and formulate strategy, whereas in countries with higher power distance, top managers may enforce a more top-down management approach. In the latter case, the negative effects of being similar on multiple characteristics may be less detrimental as the middle manager will be less engaged in divergent roles. Therefore, replications in different settings would thus be warranted.

Third, our data precluded us to test the effects of other demography dimensions, such as age and race. Although it is expected that the mechanisms specified for gender work similarly for age and race, it remains an empirical question whether they do so indeed. Also, different combinations of demography matches may have different effects (e.g. individuals that are similar in race and age may perceived as more similar than individuals similar in gender and age) on performance. Again, future work on this is warranted.

Fourth, our dependent variable, total costs per inhabitant, is an overall important indicator for municipal performance, the effects of our variables on other dependent variables, such as the implementation of specific strategies, are likely to be different. Although data limitations precluded us to specifically analyze how gender similarity would be for instance be more important for gender-affiliated strategies (e.g., such as value creation for the community), understanding when demographic similarity has an effect on which particular strategies, remains a fruitful area for future research.

Fifth, although endogeneity issues are less severe as in typical leadership studies, given that in our setting the top management team does not have full control over who will be the middle manager, endogeneity remains a potential concern. As such, we should be cautious in making causal claims based on the results.

## Conclusion

We started this paper by claiming that the strategy formation and implementation literature has underemphasized the linkages between middle and top managers [6]. Our paper sheds light on this aspect from a relational demography lens, and shows that similarity between middle and top management is associated with greater organizational performance, especially when there is similarity on one characteristic and dissimilarity on another characteristic. In so doing, our paper contributes to the middle management, relational demography, and strategy formation and implementation literatures.
